# Better Sutures

**Published:** 1895-12

**Authors:** 


It is said that the tendons found in the tail of a dog make better sutures than either catgut or kangaroo tendon, when properly prepared in sublimate.

